# Bioacoustic differentiation of calls in the chiffchaff complex

**DOI:** 10.7717/peerj.14261

**Published:** 2022-10-31

**Authors:** Maria Calviño-Cancela, Laura Piña, Julio Martín-Herrero

**Affiliations:** 1 Department of Ecology and Animal Bioloy, University of Vigo, Vigo, Spain; 2 Deptartment of Signal Theory and Communications, atlanTTic, University of Vigo, Vigo, Spain

**Keywords:** Acoustic behavior, Acoustic structure, Contact call, Forest birds, Geographic variation, Insularity, Phylloscopus, Xeno-canto

## Abstract

The chiffchaff complex is a group of common forest bird species, notorious for the number of cryptic taxa recently discovered, being a great example of speciation in action. Vocalizations have been crucial to unveil its hidden diversity. In this study we quantitatively analyze the acoustic characteristics of their calls with permutational analysis of variance, canonical variate analysis and a self-organizing map, to determine their variability and differences. We related these differences with the geographical and genetic distances between taxonomic groups, by means of Pearson correlations. We used recordings from Xeno-canto, an open database of bird vocalizations. Inter-taxa distances based on call traits were broadly consistent with geographic distances but not correlated with genetic distances. The Iberian Chiffchaff (*Phylloscopus ibericus*), presumably the most ancient lineage, was the most central in the variation space, while the Siberian Chiffchaff (*P. collybita tristis*) was the most peripheric and also very uniform, in contrast with the Canarian Chiffchaff (*P. canariensis*) highly variable, as expected by the “character release hypothesis” on islands. Calls proved to be an excellent tool, especially amenable for non-biased mathematical analyses which, combined with the wide availability of records in Xeno-canto, greatly facilitates the widespread use of this methodology in a wide range of species and geographical areas.

## Introduction

The *Phylloscopus* warblers are a large genus of insectivorous passerines, comprising more than 70 species (sensu Clements et al., 2021), and inhabiting forest habitats mostly in Eurasia and some African regions (del Hoyo, Elliot & Christie, 2006). The highest local diversity of the genus is found in the Himalayas, which is considered the center of speciation for most *Phylloscopus* species (Price, 1991; Price, Helbig & Richman, 1997). Within this genus, the ‘chiffchaff complex’ (or *Phylloscopus collybita* complex) is a group of Old World Leaf Warblers with a vast breeding range that extends across nearly the entire Palaearctic. They are especially abundant in forest ecosystems, where they can comprise up to forty per cent of all birds in some sites (Price et al., 2003). The ’chiffchaff complex’ has been historically considered as a single species (*Phylloscopus collybita*; Ticehurst, 1938) but, in the last decades, several cryptic species and a number of subspecies have been discovered in this group (Irwin et al., 2001; Rheindt, 2006; Raković et al., 2019), which makes it a great example of “speciation in action”. This, together with its abundance, has made them a great model system for studies on reproductive isolation and speciation processes (Salomon, 2002; Mahler & Gil, 2009). All members of the group have similar and inconspicuous plumages, which make them difficult to identify. The group also includes classic examples of cryptospecies, *i.e.,* species that have evolved to complete reproductive isolation but remain phenotypically indistinguishable to the human eye (Irwin et al., 2001; Olsson et al., 2005; Bickford et al., 2007). Differences in vocalizations have traditionally been used to discriminate them and have been crucial to unveil the hidden taxonomical complexity of the group. Bioacoustic together with genetic, morphological and behavioural studies (*e.g.*, differences in migration patterns) have contributed to the revelation or confirmation of the existence of cryptic taxa in this group (Helbig et al., 1996; Clement & Helbig, 1998; Raković et al., 2019).

Songbirds produce two general types of vocalizations: calls and songs. Calls are less elaborate signals than songs, they are typically short and simple, consist often of just one syllable, and have a simple frequency pattern. They also differ in their social role. While calls are produced by both sexes throughout the year, songs are typically produced by males in the breeding season. The role of songs is more restricted to reproduction and territory establishment and defence, while calls have a more diverse array of roles, including reproduction but also other functions such as warning about predators, food signalling and exchange or the maintenance of group cohesion (flock, family of mating pair) (Marler, 2004). There are many different types of calls, that can be classified according to their role in the bird life. For instance, alarm and mobbing calls are given to announce or harass predators, flight calls to coordinate the movements of the flock during flights, food calls to signal food and attract other individuals, begging calls are generally used by nestlings to press parents to provide food, and contact calls to keep in touch with other conspecifics. Contact calls, in which we focus in this study, are one of the most extensively studied types of bird calls. They are used by juveniles and adults of both sexes and encode a range of important social information such as identity, group membership, and distance (Kondo & Watanabe, 2009). Calls are geographically more uniform and seemingly stereotyped within species compared with songs (Marler, 2004), which show great variability, with individuals often having a repertoire of song types (Krebs & Kroodsma, 1980) and local dialects being a widespread occurrence among them. There are however some notable exceptions to this uniformity in calls, such as the rain call of the chaffinch (Baptista, 1990). Developmental plasticity in calls has also been shown, with experiments demonstrating vocal learning especially in parrots, but also in oscine birds, such as crossbills and chickadees (Sewall, Young & Wright, 2016 and references therein). Nevertheless, according to Marler (2004), there is little doubt about the innate nature of the acoustic structure of most calls. Despite the paucity of studies focused on calls (in contrast to songs) some studies suggest that contact calls might be under sexual selection and have a role as a prezygotic isolation barrier (Husemann, Ulrich & Habel, 2014).

The recognition of vocalizations, songs and calls, is a key mechanism for mate choice in birds (Nowicki & Searcy, 2005). The origin of vocalization divergence is related to three main processes: stochastic processes such as genetic drift and mutation (Kimura, 1983; Lynch & Hill, 1986); sexual selection, with signals evolving to maximize attractiveness for potential mates (Ryan & Rand, 1993); and ecological adaptation (often termed acoustic adaptation in this context), with frequency and time parameters of vocalizations responding to selective pressures related to the sound transmission properties of habitats (*e.g.*, structure of vegetation), minimizing signal degradation and maximizing conspicuousness against background noise and interference, resulting in signal divergence across ecological gradients (Morton, 1975; Hansen, 1979; Patten et al., 2004). Geographic isolation and differing environmental conditions may drive signal differentiation across distribution ranges. Over time, vocalizations diverge between allospecies, which contribute to premating isolation (Salomon, 1989; Päckert et al., 2009; Price, 2010). Vocalization divergence has been mostly studied in songs (Mahler & Gil, 2009; Tietze et al., 2015), while call differentiation has received less attention. However, calls may differ clearly between species and may also have an important role in species recognition, *e.g.*, Helbig et al. (1996). Phylogenetic signal of bird vocalizations is considered to be high, particularly of innate vocalizations (McCracken & Sheldon, 1997; Päckert et al., 2003).

In this study we aim to quantitatively analyze the acoustic characteristics of the calls of the chiffchaff complex, in order to study the similarity/distance relationships between taxonomic groups, the degree of variability within and between them, as well as to relate them with genetic distances revealed in previous studies as well as with geographical distances, by means of correlations. In contrast to songs, calls have a simpler acoustic structure, which make them more amenable to mathematical analyses. We used objective acoustic features of the calls, selected according to their robustness, avoiding subjective discrimination based on perceived differences between calls. The call recordings used were taken from Xeno-canto (Xeno-canto Foundation, 2022), an open database of recordings of bird vocalizations, which allowed us to sample the calls from the main groups of the chiffchaff complex covering a wide geographical range.

## Materials and Methods

### Taxonomy of the chiffchaff complex

**Figure 1:**
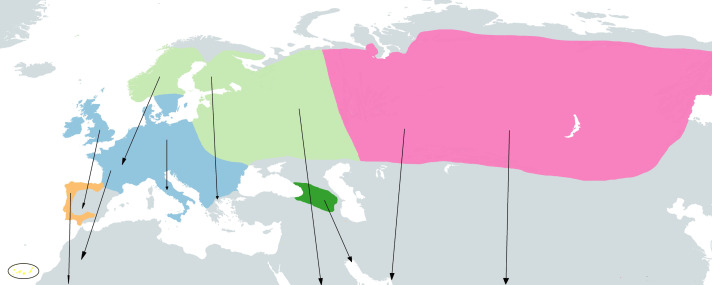
Approximate geographical ranges of the studied *Phylloscopus* groups. Subspecies *collybita*, *abietinus* and *tristis* of *Phylloscopus collybita* are represented in blue, light green and pink, respectively, *Phylloscopus ibericus* in orange, *Phylloscopus canariensis* in yellow, highlighted by a black circle due to the small size of the islands, and *Phylloscopus sindianus lorenzii* in dark green. Arrows represent migrations from reproductive to wintering areas. Based on Shirihai & Svensson (2018) and Raković et al. (2019).

The taxonomy of the group here described is based on the IOC World Bird List (Gill, Donsker & Rasmussen, 2022). Four cryptic species have been described within the so called ’chiffchaff complex’: Common Chiffchaff (*Phylloscopus collybita* Vieillot, 1817), Iberian Chiffchaff (*P. ibericus* Ticehurst, 1937), Canary Islands Chiffchaff (*P. canariensis* Hartwig, 1886) and Mountain Chiffchaff (*P. sindianus* W.E. Brooks, 1880) (Raković et al., 2019; Crochet & Joynt, 2015). In addition, the Common Chiffchaff is divided into six subspecies (Dickinson & Christidis, 2014; ITIS, 2022): *P. c. collybita* (Vieillot, 1817), breeding in Western, Central and Southern Europe, wintering in Southern Europe, North Africa and the Middle East (see Fig. 1); *P. c. abietinus* (Nilsson, 1819), breeding in Northern and Eastern Europe), it winters in South Europe, North Africa, Middle East, North Arabia, Persian Gulf and Southwest Iran (see Fig. 1); *P. c. tristis* (Blyth, 1843) (Siberian Chiffchaff), breeding in easternmost Russia, Siberia, North Kazakhstan, North Mongolia, coming into contact with *P. c. abietinus* in West Russia (Shipilina et al., 2017), and wintering in South and Southeast Asia, predominantly India, also South Central Asia, Middle East, Turkey and local Southern Europe (see Fig. 1); *P. c. brevirostris* (Strickland, 1837), breeding in western and northern Anatolia (Turkey); *P. c. caucasicus* (Loskot, 1991), breeding in the Greater and Lesser Caucasus, East of *P. c. brevirostris* range at lower elevations south to Armenia; and *P. c. menzbieri* (Shesteperov, 1937), believed to be restricted to the mountains of northeastern Iran and Turkmenistan. The Iberian Chiffchaff (*P. ibericus*) breeds in the Iberian Peninsula and winters in subsaharian Africa (Pérez-Tris, Ramírez & Tellería, 2003; Catry et al., 2005) (see Fig. 1). Two subspecies have been described for the Canary Islands Chiffchaff (*P. canariensis*) (ITIS, 2022), *P. canariensis exsul* Hartert, 1907, from the island of Lanzarote (NE Canary Islands), which is believed to be extinct, and *P. canariensis canariensis* (Hartwig, 1886), from other islands of the Canary archipelago: La Palma, Hierro, Gomera, Tenerife and Gran Canaria (see Fig. 1). The Mountain Chiffchaff (*P. sindianus*) is restricted to mountains and includes two subspecies (ITIS, 2022), *P. sindianus sindianus* (W.E. Brooks, 1880), from the extreme west of China (SW Xinjiang) to North Pakistan and North India and *P. sindianus lorenzii* (T. Lorenz, 1887), from Southwest Asia, in East Turkey to Caucasus, Transcaucasia and Northeast Iran (see Fig. 1).

### Data collection

Call recordings were obtained from Xeno-canto (Xeno-canto Foundation, 2022), an open-access repository run by the Xeno-canto Foundation (Holland) for sharing recordings of bird sounds worldwide and to improve their knowledge. This allowed us to analyze the characteristics of calls from across a wide geographical range. Recordings were selected as to be representative of the geographical range of the sample in Xeno-canto, subject to their availability. We selected only common contact calls, not including alarm calls, calls used while carrying food to the nest, calls of juvenile individuals or alternative calls, such as those commonly referred to as sweeoo calls (Lindholm, 2014). We registered the following data from each recording: recorder name, date (year-month-day), time of day (hour:min), geographical coordinates (latitude, longitude), locality and country, catalogue number, and recorder comments. For selection we took into account the sound quality, the background noise, and the number of calls recorded. When several recordings were captured by the same recorder in the same locality and whithin a short span of time, we took into account the likelihood of them corresponding to the same individual bird, thus we selected only the recording with most calls and best recording quality. All recordings were in mp3 format, with a sampling rate of 44.1 kHz in most cases, or higher (48 kHz), except one case sampled at 32 kHz.

**Figure 2:**
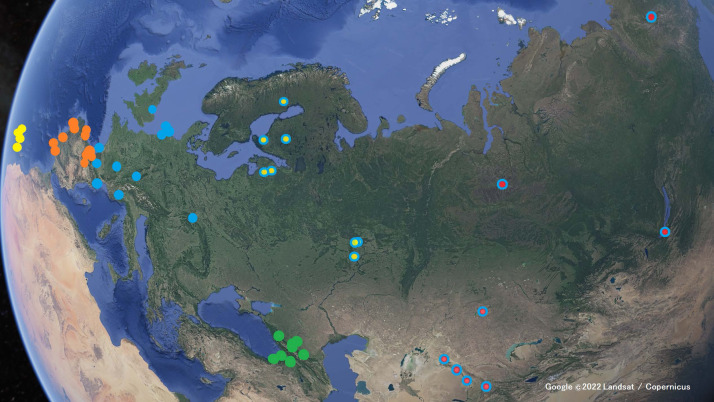
Locations where the Xeno-canto recordings were taken. Yellow markers for *Phylloscopus canariensis*; orange markers for *P. ibericus*; blue markers for the *P. collybita* subspecies (*P. c. collybita*: solid blue markers; *P. c. abietinus*: with yellow center; and *P. c. tristis*: with red center); and green markers for *P. sindianus lorenzi*. (Map data: Google ©2022 Landsat / Copernicus.)

We compiled a total of 82 recordings with 735 calls of individuals belonging to the following taxa (Table 1; see map in Fig. 2): *Phylloscopus collybita collybita*, *P. collybita abietinus*, *P. collybita tristis*, *P. ibericus*, *P. sindianus lorenzii*, and *P. canariensis*. For the latter, we sampled individuals from four of the Canary Islands (Tenerife, Tnf; Gran Canaria, GrC; La Gomera, Gom; La Palma, Pal). We did not include subspecies *P. c. brevirostris*, *P. c. caucasicus* and *P. c. menzbieri* in this study due to the scarce number of call recordings on Xeno-canto (three, two and one, respectively). We also did not include *P. sindianus sindianus* due to the lack of recordings on Xeno-canto. With the criteria above, each recording in Table 1 should correspond to one individual. In some cases, calls from different individuals could be distinguished in the same recording (by their different amplitudes, related to distance to the sound recorder), wherein we selected only the calls with similar amplitudes, corresponding to a single individual.

**Table 1:** List of taxa and, in the case of *Phylloscopus canariensis*, populations (islands) sampled, with the recording codes as taken from Xeno-canto (XC), and the number of calls analyzed.

**Taxa/population**	**XC code**	**# calls**	**Taxa/population**	**XC code**	**# calls**
*Phylloscopus canariensis*				194589	11
La Gomera Island	215106	11		201507	7
	215111	11		337194	11
	215124	11		347406	3
	460045	11		347407	4
Gran Canaria Island	196065	5		366263	7
	290216	11		669878	11
	398572	2	Subsp. *tristis*	120364	4
	458953	2		145063	11
La Palma Island	122550	11		182891	11
	270057	11		183072	11
	270062	5		183309	11
	270067	11		184005	9
Tenerife Island	45371	11		431105	5
	45372	11		484307	4
	134417	11		676168	12
	347437	7		676186	11
	349326	11	*Phylloscopus ibericus*		
	349339	11		150191	5
	354852	4		375626	11
	363079	4		378186	11
	366489	11		409146	4
	367183	8		409243	11
	497618	5		415842	11
*Phylloscopus collybita*				428200	11
Subsp. *collybita*	39335	11		483346	11
	112457	11		484733	8
	113397	6		553339	11
	149722	11		564974	11
	165231	11		577383	11
	175849	11		582751	5
	235074	11		583671	3
	374622	11	*Phylloscopus sindianus lorenzii*		
	429244	11		139517	10
	492107	8		193395	4
	565767	11		197894	12
	570469	12		197961	6
	608206	4		197962	11
Subsp. *abietinus*	133094	11		217386	11
	161815	9		281861	11
	189227	11		340597	5
	193634	11		341076	11
	194082	11		356976	9

The number of individuals per taxa ranged from 10 for *P. s. lorenzii* to 23 for *P. canariensis* (Table 1). The number of calls per individual averaged nine, with 11 calls for 59% of individuals (mode), 12 was the maximum, and two the minimum, in two of the four *P. canariensis* individuals from Gran Canaria, due to the low availability of recordings in this island.

### Sound processing and analysis

Stereo recordings were converted to mono with Audacity^®^ (Audacity Team, 2021) prior to bioacoustic analyses. Then calls were analyzed with Raven Pro 1.6.1. (Center for Conservation Bioacoustics, 2019; Charif, Waack & Strickman, 2010). For each call, we selected the fundamental frequencies and discarded the overtones. To characterize the calls, we used 13 acoustic parameters (see Table 2 for a description), focusing on robust signal characteristics that take into account the energy in the segment of the spectrogram to be analyzed, thus minimizing the possible bias introduced by the observer when delimiting the boundaries of the call in the spectrogram. The variables used describe frequency, time, and shape features of the sound signals.

**Table 2:** Features used in this study to characterize the calls. The abbreviations used by the Raven software (Charif, Waack & Strickman, 2010) have been added for reference. All frequencies in Hz and time lapses in seconds.

**Abbreviation**	**Description**
Center Freq	Center Frequency: The one dividing the selection into two intervals of equal energy.
Freq 5%	Frequency 5%: The one dividing the selection into two intervals containing 5% and 95% of the energy.
Freq 25%	Frequency 25%: The one dividing the selection into two intervals containing 25% and 75% of the energy.
Freq 75%	Frequency 75%: The one dividing the selection into two intervals containing 75% and 25% of the energy.
Freq 95%	Frequency 95%: The one dividing the selection into two intervals containing 95% and 5% of the energy.
Max Freq	Max or Peak Frequency: The one at which the maximum power occurs within the selection.
PFC Max Freq	Peak Frequency Contour Max Frequency: Maximum frequency of the series of peak frequencies for each spectrogram slice.
PFC Min Freq	Peak Frequency Contour Min Frequency: Minimum frequency of the series of peak frequencies for each spectrogram slice.
PFC Num Inf Pts	Peak Frequency Contour Number of Inflection Points: Number of times the slope changes sign in the series of differences between each pair of adjacent elements in the peak frequency contour.
Dur 50%	Duration 50%: Time between the point dividing the selection in two intervals containing 25% and 75% of the energy and the point dividing the selection in a first interval containing 25% of the energy and a second containing 75% of the energy.
Dur 45% ante^1^	Duration 45% anterior: Time between the point dividing the selection in two intervals containing 5% and 95% of the energy and the point in time dividing the selection in two intervals of equal energy (50% each).
Dur 45% post^1^	Duration 45% posterior: Time between the point dividing the selection in two intervals of equal energy (50% each) and the point in time dividing the selection in two intervals containing 95% and 5% of the energy.
Dur 90%	Duration 90%: Time between the point dividing the selection in two intervals containing 5% and 95% of the energy and the point dividing the selection in a first interval containing 95% and a second containing 5% of the energy.

**Notes.**

1 Not directly provided by the Raven software.

### Data analysis

Permutational analysis of variance (PERMANOVA) was used to determine differences between the *Phylloscopus* groups studied (nine groups, as described in the Data collection section) and within the groups (*i.e.,* between the individuals sampled, nested within the group) in regard to the bioacoustic characteristics of the calls. We also estimated their variance components, *i.e.,* the contribution of the variation between and within groups to the overall variability of the data. Pairwise differences among groups (species and subspecies) were calculated using a multivariate analogue to the univariate *t* statistic, calculating pseudo- *t* as the square root of pseudo- *F* between each pair of groups. The *P*-values of both the pseudo- *F* (in the main PERMANOVA analysis) and the pseudo- *t* in the pairwise tests, were obtained using permutations. PERMANOVA analyses were performed with PRIMER 6.1.12 (Clarke & Gorley, 2006) with the PERMANOVA + 1.0.2 add-on Anderson, Gorley & Clarke (2008). They were based on Euclidean distances using type III(partial) fixed effects sum to zero for mixed terms and permutation of residuals under a reduced model using 9999 permutations.

We used canonical discriminant analysis (CDA; also termed canonical variate analysis —CVA; Campbell & Atchley, 1981; Matthew et al., 1994) to analyze the group structure of our multivariate data, by means of the CVA procedure within the statistical package GenStat 7th ed. (Payne et al., 2003). This is an efficient method to analyze multidimensional data having some degree of correlation (redundancy), by reducing data redundancy and finding the canonical discriminant functions (linear combinations of the variables) that maximize the separation (discrimination) among the groups. Since the variates are expressed in different scales and had different amounts of variance, we first standardized them by subtracting from each value (*x*) the minimum value (min) and dividing by the range (max –min):(*x*-min)/(max–min). The canonical discriminant analysis was performed with the standardized values. In order to better understand the similarity/distance relationships between taxonomic groups, we graphed them in a canonical variates biplot that shows the spatial arrangement of the calls of the different *Phylloscopus* taxa studied in the canonical variates space, according to their acoustic features. We analyzed the relationship of inter-taxa CDA distances (Mahalanobis distances) with (i) genetic distances and (ii) geographic distances using Pearson correlations. Inter-taxa genetic distances were obtained from Helbig et al. (1996) and Raković et al. (2019). Geographic distances were calculated from the geographic centers of the distribution range of each taxa.

In addition, we mapped all the calls acoustic features to a two-dimensional manifold using a discrete self-organizing map (SOM) (Kohonen, 1982; Kohonen, 1990; Aymerich et al., 2016) with 15 × 10 nodes or neurons. This is an unsupervised machine learning technique useful to represent a high dimensional data as a low-dimensional graph (two-dimensional in this case), making high dimensional data easier to visualize while preserving the topological structure of the data. It is a type of artificial neural network trained using competitive learning. Once the SOM was built, we fitted a two-dimensional Gaussian to the calls of each of the *Phylloscopus* groups by means of Singular Value Decomposition of the mappings to the 2D manifold of all calls in the group, and placed them over the map (Calviño-Cancela & Martín-Herrero, 2016), thus providing a visual representation of the topological relationships among the groups in their original, 13D feature space.

## Results

### PERMANOVA analysis

The PERMANOVA analysis (Table 3) showed that calls differed significantly regarding the bioacoustic parameters analyzed, both between the groups and within the groups (between the individuals sampled in different areas within the distribution range). The analysis yielded similar results (Pseudo- *F* = 11.34, *P* = 0.001 between groups and Pseudo- *F* = 22.37, *P* = 0.001 within groups) when the populations in different islands within *P. canariensis* were combined. The variance in the characteristics of the calls accounted for by the differentiation between groups (42.5% of the total variance) was similar to that within groups (39.2%) The residual variance, corresponding to variation between observations not accounted for by the factor considered, in this case corresponding to calls within the sampled individuals, contributed another 18.4%. The relative contributions of the differentiation between groups and within groups varied when the islands within *P. canariensis* were combined, with differences between groups amounting to 36.9% of the total variance, and within groups to 44.6%. Note that the individuals sampled for each group do not coexist but were sampled from distant areas, so that the variance within groups encompasses regional, habitat and individual variance.

**Figure 3:**
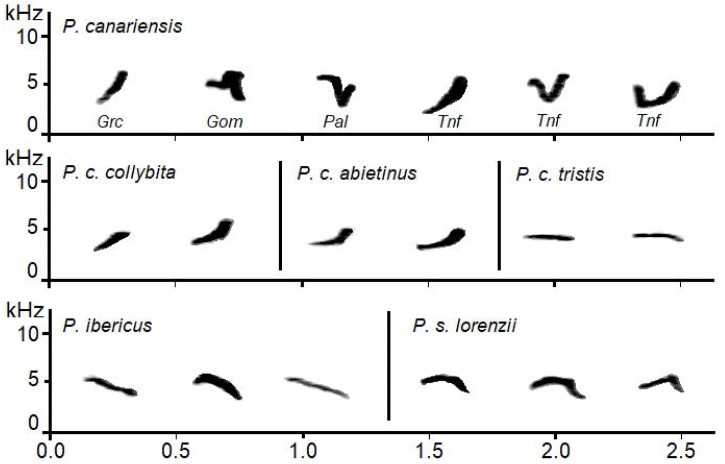
Examples of sonograms of the calls of the studied *Phylloscopus* groups. For *Phylloscopus canariensis* examples from different Canary Islands are shown with abbreviations as follows: Grc for Gran Canaria, Gom for La Gomera, Pal for La Palma, and Tnf for Tenerife.

Pairwise differences among species and subspecies were all significant (*t* ≥ 2.4, *P* ≤ 0.002) except for subspecies *P. c. collybita* and *P. c. abietinus*, whose calls were not statistically different (*t* = 0.44, *P* = 0.972) Despite the small sample size obtained for *P. canariensis* for some of the islands, some of the differences between islands were significant, specifically Gomera showed significant differences with Gran Canaria and Tenerife, and La Palma with Tenerife (*t* = 2.2, 2.1, 1.8 and *P* = 0.041, 0.026, 0.007, respectively). See Fig. 3 for the spectrograms of the characteristic calls of each *Phylloscopus* group studied.

**Table 3:** Results of the permutational analysis of variance for the effects of the explanatory variables on the bioacoustic characteristics of the calls.

**Source of variation**	**df**	**SS**	**Pseudo-F**	**P(perm)**	**% of variance**
Group	8	86.51	10.177	0.001	42.5
Within Group	73	89.59	19.890	0.001	39.2
Residual	653	40.29			18.4
Total	734	227.03			100

### Canonical Discriminant Analysis

**Figure 4:**
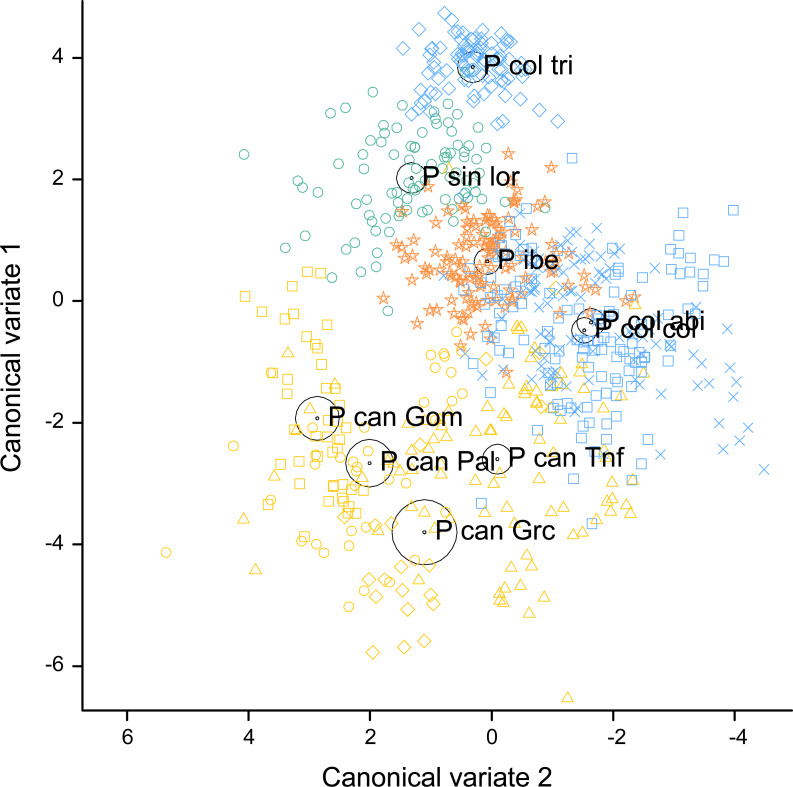
Canonical discriminant analysis biplot showing the spatial arrangement in the canonical variate space of the calls sampled from the studied *Phylloscopus* groups according to their acoustic features. Canonical variate 1 and 2 correspond with the vertical and horizontal axis, respectively. Group centroids and concentric 95% confidence regions are displayed. Abbreviations are as follows: P can for *Phylloscopus canariensis*, with Gom, Pal, Grc, and Tnf referring to the Canary Islands sampled (La Gomera, La Palma, Gran Canaria, and Tenerife); P col tri for *P. collybita tristis* (in blue diamonds), P col col for *P. c. collybita* (in blue squares) and P col abi for *P. c. abietinus* (in blue crosses); P sin lor for *P. sindianus lorenzii* (in green circles) and P ibe for *P. ibericus* (in orange stars).

The two first canonical discriminant functions explained 84.9% of the total variance (60.2% the first one and 24.7% the second). This percentage reached 91.7% when the *P. canariensis* of different islands were combined, with 72.3% explained by the first and 19.4% by the second canonical discriminant function. Shape, time and frequency features were all represented among the variables with the largest contributions to the canonical variates. PFC Num Inf Pts and Dur 50% were the variables with the highest loadings (Table 4) in the first canonical discriminant function, followed by Dur 45% post, PFC Max Freq, and Center Freq, while Max Freq and Dur 45% post had the highest loadings in the second canonical discriminant function. As indicated by the canonical loadings (Table 4), the first canonical discriminant function, represented as the vertical axis in Fig. 4, increases with increasing Dur 50%, Center Freq and PFC Max Freq (positive and relatively large loadings) and with decreasing PFC Num Inf Pts and Dur 45% post (negative and relatively large loadings), whereas the second canonical discriminant function, represented as the horizontal axis in Fig. 4, increases mainly with decreasing Max Freq and Dur 45% post.

**Table 4:** Latent vectors (loadings) showing the contributions of each acoustic feature (Table 2) to the canonical variates (canonical discriminant analysis).

**Acoustic feature**	**First axis**	**Second axis**
Center Freq	3.415	−2.231
Freq 25%	1.171	0.418
Freq 5%	0.819	−0.992
Freq 75%	0.412	−0.414
Freq 95%	1.990	−0.52
Max Freq	0.583	−5.698
PFC Max Freq	3.315	−0.147
PFC Min Freq	−0.154	−0.445
PFC Num Inf Pts	−9.385	0.811
Dur 45% ante	−0.766	1.483
Dur 45% post	−4.004	−3.488
Dur 50%	6.399	−2.038
Dur 90%	−0.699	−0.666

**Figure 5:**
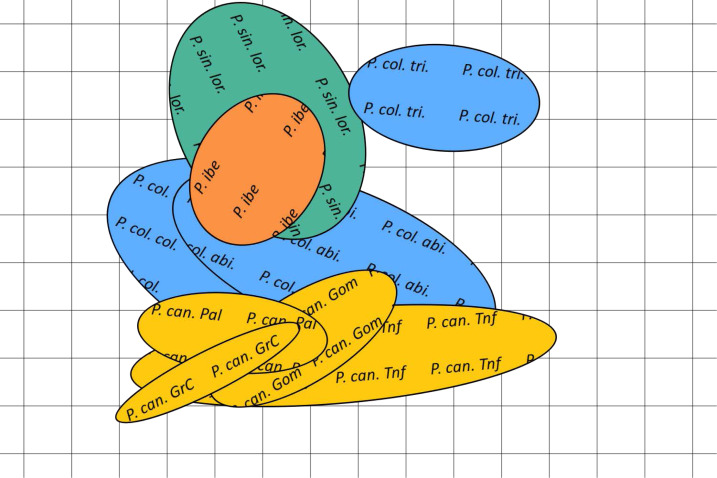
Bidimensional SOM mapping of the calls sampled from the studied *Phylloscopus* groups according to their acoustic features. The coloured ellipses correspond to a Gaussian fit by singular value decomposition of the mapping to the two dimensional manifold of each call for each of the *Phylloscopus* groups studied. In yellow *Phylloscopus canariensis* from different islands, in blue *P. collybita* subspecies (*collybita*, *abietinus* and *tristis*), in green *P. sindianus lorenzi* and in orange *P. ibericus*. Abbreviations as in Fig. 4.

**Figure 6:**
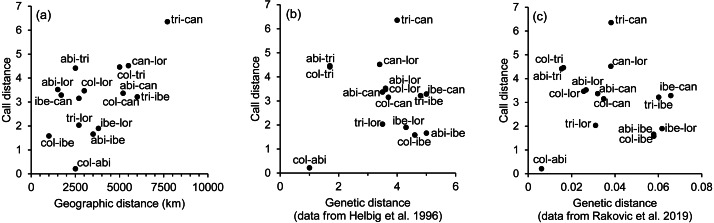
Scatterplots showing the relationship between call, geographic and genetic distances. Association between the inter-taxa call distances, as revealed by the canonical discriminant analysis (Mahalanobis distances), and (A) geographic distances, (B) genetic distances obtained from Helbig et al. (1996), and (C) genetic distances obtained from Raković et al. (2019). Taxa abbreviations are as follows: abi, col and tri for *Phylloscopus collybita* subspecies *abietinus*, *collybita* and *tristis*, respectively; can for *P. canariensis*; ibe for *P. ibericus* and lor for *P. sindianus lorenzii*.

The spatial arrangement of the *Phylloscopus* complex shown in the Canonical Discriminant Analysis biplot (Fig. 4) was coherent with the mapping of the spectral features to a 2D manifold performed by the SOM (Fig. 5). It shows *P. canariensis* calls from the different Canary Islands grouped in the lower left half of the biplot while the calls of all the rest of groups (*P. sindianus lorenzii*, *P. ibericus* and subspecies or populations of *P. collybita*) appear in the other half of the graph (Fig. 4). The Iberian Chiffchaff, *P. ibericus*, occupies the most central position in the variation space (minimum average distance with the other species and subspecies, and minimum variance). In contrast, the Siberian Chiffchaff (*P. c. tristis*) forms a rather compact and distinct group in the top of the biplot, with the largest average inter-group distance with all the rest and maximum variance. The greatest distances were found between *P. c. tristis* and the *P. canariensis* from the different islands sampled (Table 5; Figs. 4 and 5). Within *P. collybita*, subspecies *tristis* was the most distant to the nominate subspecies *collybita*, while it was closer to *P. s. lorenzii*, and then to *P. ibericus* (Table 5; Figs. 4 and 5). In contrast, *P. c. collybita* and *P. c. abietinus* were the closest groups (Table 5), with the calls from these two subspecies intermixed in Fig. 4 and group centroids not statistically significant (overlapped 95% confidence intervals; Fig. 4, see also the overlap in Fig. 5), which reaffirms the results obtained with the PERMANOVA. The arrangement of *Phylloscopus* taxa in Fig. 4 has some resemblance to their geographical distribution, with inter-group distances according to the Canonical Discriminant Analysis showing a moderate correlation with geographic distances (*r* = 0.59, Pearson correlation; Fig. 6A). However, inter-group distances showed weak to negligible correlation with the genetic differences shown in previous studies (*r* =  − 0.02 with the data from Helbig et al. (1996) and −0.12 with the data from Raković et al. (2019), Pearson correlation; see Figs. 6B and 6C). The greater discrepancies between call and genetic distances occurred for the distances between *P. c. collybita* or *P. c. abietinus* with *P. c. tristis* and with *P. ibericus* (Figs. 6B and 6C), with relatively larger call than genetic distances for the differences with *tristis* and larger genetic than call distances for the differences with *ibericus*.

**Table 5:** Inter-group distances according to the canonical discriminant analysis. Abbreviations as in Fig. 4.

	**P can Gom**	**P can GrC**	**P can Pal**	**P can Tnf**	**P col abi**	**P col col**	**P col tri**	**P ibe**	**P sin lor**
**P can Gom**	0								
**P can GrC**	2.571	0							
**P can Pal**	1.131	1.451	0						
**P can Tnf**	3.036	1.697	2.104	0					
**P col abi**	4.771	4.406	4.317	2.726	0				
**P col col**	4.623	4.234	4.153	2.555	0.172	0			
**P col tri**	6.318	7.691	6.732	6.460	4.628	4.702	0		
**P ibe**	3.804	4.571	3.841	3.255	1.98	1.957	3.205	0	
**P sin lor**	4.245	5.828	4.739	4.833	3.788	3.787	2.085	1.852	0

The arrangement of *P. canariensis* from the different islands showed that, despite some degree of intermixing, there was differentiation between islands, and group centroids appeared significantly separated, although with relatively short intergroup distances. The most similar calls among *P. canariensis* islands, according to the discriminant analysis, were those of La Gomera (Gom) and La Palma (Pal), and of La Palma (Pal) and Gran Canaria (GrC), followed by those of Gran Canaria (GrC) and Tenerife (Tnf) (differences between these populations were not significant in the PERMANOVA analysis, as previously described). Tenerife was the closest to *P. c. collybita* and *P. c. abietinus* (Table 5; Fig. 4).

## Discussion

As far as we know, this is the first study with a quantitative analysis of the acoustic differences in the calls of the chiffchaff complex. This quantitative analysis allowed us to determine the relationships within this complex according to the characteristics of their calls as well as to determine the degree of variability within groups (species, subspecies and populations) and examine its variance components.

We found significant differentiation in the acoustic characteristics of the calls of the chiffchaff complex. The spatial arrangement obtained with the canonical analysis was coherent with that obtained with the SOM mapping. There was a good match between the two methods in the relative positions of the different groups, distance patterns and neighbourhood relations, despite the fundamental differences between the two procedures, which adds confidence in the results. The canonical variates biplot depicts a gradual change between the different *Phylloscopus* groups analyzed in regard to the acoustic features of the calls. Their arrangement in the acoustic variation space has some resemblance to their geographical distribution, with inter-group distances broadly consistent with geographic distances between groups (*r* = 0.59). However, their relationship with genetic distances (Helbig et al., 1996; Raković et al., 2019) was very weak. In fact, genetic distances showed weak to negligible correlation with geographic distances (*r* = 0.03 with the data from both Helbig et al. (1996) and Raković et al. (2019); Pearson correlation).

Calls, as well as songs and other phenotypic traits, are not neutral characters, but are subject to selection, in contrast to microsatellites or mitochondrial haplotypes that are used to measure genetic distances. The effect of selection may contribute to blur the relationship between call and genetic distances, as well as with geographic distances. Helbig et al. (1996) also noted contrasting patterns between phenotypic and genetic characters in the chiffchaff complex. In regard to geographic distances, we have to bear in mind that the current distribution range of these taxa do not necessarily reflect their past distribution, as they have experienced successive range expansions and retractions, following the changes in forest distribution throughout thousands of years of climate changes, especially during the Pleistocene glaciations (Holm & Svenning, 2014). A better knowledge of past distribution should shed more light on the effect of isolation by distance on taxa differentiation within this species complex.

The most central position in the variation space (minimum variance in the distances to other groups) was occupied by *P. ibericus*, presumably the most ancient lineage within the chiffchaff complex (Helbig et al., 1996; Raković et al., 2019). This central position means that the changes needed to go from the characteristics of *P. ibericus* calls to those of all other taxa are relatively small in all cases(the smallest overall), as compared to the changes needed to go from any of the species or subspecies analyzed to any of the rest. This central position, however, does not match its present geographic location, which is rather peripheral in relation to the whole chiffchaff complex: In particular, *P. s. lorenzi* and *P. c. tristis* have more similar calls to *P. ibericus* than to *P. c. abietinus* and *P. c. collybita*, which are geographically closer. Genetic analyses also show a counter intuitive result regarding *P. ibericus*, as it formed the sister group of subspecies *sindianus* and *lorenzii* of *P. sindianus*, being closer to them than to *collybita* or *canariensis* (Helbig et al., 1996). Again, the complex history of range expansions and retractions in the past might have contributed to blur the relationship with current geographic patterns.

In contrast to the central position of *P. ibericus*, the calls of *P. c. tristis* were the most peripheral, and this was the subspecies showing the most differentiated calls within *P. collybita*, being more similar to *P. s. lorenzi*, a different species. The subspecies *tristis* is also the most distant geographically to most of the other species or subspecies, particularly to *P. canariensis*, with which it showed the greatest differences in call traits. The higher similarity of *P. c. tristis* with *P. s. lorenzi* than with other *P. collybita* subspecies is consistent with the divergence patterns shown by song and morphological (brownish plumage) characters (Helbig et al., 1996; Ilina et al., 2021) but contrasts with those according to molecular traits, that show *P. c. tristis* as closer to other *P. collybita* subspecies than to *P. s. lorenzi* (Helbig et al., 1996; Raković et al., 2019). Genetic analyses (Helbig et al., 1996; Raković et al., 2019) agree however with analyses based on phenotypic characters (this study; Helbig et al., 1996; Ilina et al., 2021) in showing *P. c. tristis* as the most differentiated subspecies, which led (Raković et al., 2019) to argue that it may represent a separate species or at least may be in a stage of incipient speciation or semispecies status (Helbig et al., 2002). In support to its subspecies status is the mass hybridization and significant gene flow that occurs between *P. c. tristis* and *P. c. abietinus* in secondary contact zones, as well as the responses of territorial males to the playback song of the other taxon (Shipilina et al., 2017; Marova et al., 2017). According to Raković et al. (2019), the diversification within *P. collybita* began with the separation of this group, *tristis*, from the common ancestor during the Ionian stage of the Pleistocene, approximately 290 kyr BP. Its taxonomic status is still debated and, although it is considered as a subspecies of Common Chiffchaff (*P. c. tristis*) by the IOC World Bird List (Gill, Donsker & Rasmussen, 2022), some consider it as a full species (*P. tristis*; *e.g.*, del Hoyo & Collar (2017); Shirihai & Svensson (2018)). In addition to being the most distinct, the calls of *tristis* were also remarkably uniform, especially considering their large geographical range. This contrasts with the much higher variability shown by the nominate subspecies *P. c. collybita* or by *P. c. abietinus*, with a much smaller geographical range. This relative uniformity of *P. c. tristis* calls contrasts with the variability shown in their songs (Helbig et al., 1996) and with its relative higher genetic diversity compared to *P. c. abietinus*, as revealed by Shipilina et al. (2017).

Also noteworthy was the variability in the calls of *P. canariensis*, as they occupy almost half of the whole variation space in Fig. 4, despite the reduced geographical range of the species. Some differentiation between islands was apparent in the biplot, which has been also revealed at the mitochondrial genetic level (Helbig et al., 1996), with remarkable haplotype diversity together with genetic differentiation between islands, with the occurrence of derived haplotypes on different islands. There was also remarkable variability within islands, between the individuals sampled and between calls within individuals. This agrees with the Character Release Hypothesis (Naugler & Ratcliffe, 1994). Island ecosystems are characterized by decreased species diversity compared to mainland communities (MacArthur & Wilson, 1967), and thus reduced interspecific competition, which in turn may lead to character release and broader ecological niches (including acoustic niches), favoring higher behavioural diversity within species, as part of the so-called island syndrome. Given the importance of vocalizations for species recognition and mate choice in birds (Nowicki & Searcy, 2005), selection should favor species distinctiveness and limit within-species variation, in order to improve mate identity (Podos, Huber & Taft, 2004). In islands, however, where species diversity is low, selection for species distinctiveness is relaxed (character release), and vocalizations are expected to be more variable compared to mainland (Morinay et al., 2013).

Island differentiation in call notes of *P. canariensis* was already pointed out by Henning, Schottler & Martens (1994) and further studied by Naguib, Hammerschmidt & Wirth (2001). In Naguib, Hammerschmidt & Wirth (2001), they analyzed the call structure of *P. canariensis* (regarded in their study as a subspecies of *P. collybita*: *P. collybita canariensis*) in two islands of the Canaries, in habitats with contrasting vegetation structure, from scarcely vegetated agricultural habitats to dense forests. They found call structures differing between the two islands but also between individuals as well as microgeographic variation, but no differences between habitats, in contrast to the acoustic adaptation hypothesis (Naguib, Hammerschmidt & Wirth, 2001).

The calls of *P. c. collybita* were also highly variable, and thoroughly intermixed with those of *P. c. abietinus*. These two subspecies are morphologically very similar as well, but despite these similarities, they are clearly genetically differentiated (cytochrome b sequence divergence has been estimated as 1.0%; Helbig et al., 1996). Although there is evidence of mitochondrial gene flow between them, this seems to occur at a low rate (*abietinus* genotype was found in 3.8% of Central European *collybita* individuals; Helbig et al., 1996). These subspecies have become recently in contact in southern Scandinavia, due to the northward expansion of *collybita* (Hansson, Bensch & Brännström, 2000). Significant morphological differentiation was found in this area between the northern (*abietinus*) and southern(*collybita*) populations and, despite song similarity, playback experiments revealed that males react more strongly to males of their own subspecies than to the song of the other subspecies, which means that they distinguish the songs and react differently (Hansson, Bensch & Brännström, 2000). There are also differences in habitat choice, with *collybita* breeding mainly in deciduous forests while *abietinus* prefers coniferous forests (Hansson, Bensch & Brännström, 2000). Thus, behavioural characteristics such as vocalizations and habitat choice help to limit hybridization and gene flow in the contact zone. However, differences in habitat choice did not translate in differentiation of call characteristics associated to habitat selection (according to the acoustic adaptation hypothesis; Morton, 1975). This is not unexpected, since the effect of habitat structure on the acoustic properties of bird vocalizations seems to be weak, even when comparing habitats with clearly contrasting vegetation structure (densely vegetated compared to herbaceous habitats), as shown in a meta-analysis by Boncoraglio & Saino (2007). Since all the *Phylloscopus* taxa analyzed are typically forests birds, a strong effect of habitat is not to be expected, according to these previous studies.

## Conclusions

Our study provides the first quantitative assessment of call differentiation and patterns of variation in relation to geographic and genetic distances in the *Phylloscopus* complex. We found striking variability in the calls of *P. canariensis*, by far the most variable in regard to its geographical range, which agrees with the character release hypothesis associated to the reduced interspecific competition in islands. Our results are consistent with previous genetic studies in showing the distinctiveness of *P. c. tristis*, supporting its full species status or being in a stage of incipient speciation. Calls proved to be especially amenable for the application of non-biased mathematical analysis. This combined with the wide availability of records in an open-access repository such as Xeno-canto, greatly facilitates the widespread use of this methodology in a wide range of species and geographical areas, providing another example of the usefulness of citizen science. In conclusion, calls are a valuable tool to promote our knowledge in avian diversification processes in the near future.

## Additional Information and Declarations

### Data Availability

The following information was supplied regarding data availability: The raw data is available in the open-access repository Xeno-canto (https://xeno-canto.org/). The codes of the specific records used in this study are available in Table 1.
